# A Ready-to-Use Grading Tool for Facial Palsy Examiners—Automated Grading System in Facial Palsy Patients Made Easy

**DOI:** 10.3390/jpm12101739

**Published:** 2022-10-19

**Authors:** Leonard Knoedler, Maximilian Miragall, Martin Kauke-Navarro, Doha Obed, Maximilian Bauer, Patrick Tißler, Lukas Prantl, Hans-Guenther Machens, Peter Niclas Broer, Helena Baecher, Adriana C. Panayi, Samuel Knoedler, Andreas Kehrer

**Affiliations:** 1 Department of Plastic, Hand and Reconstructive Surgery, University Hospital Regensburg, 93053 Regensburg, Germany; 2 Department of Oral and Maxillofacial Surgery, University Hospital Regensburg, 93053 Regensburg, Germany; 3 Department of Surgery, Division of Plastic Surgery, Yale New Haven Hospital, Yale School of Medicine, New Haven, CT 06510, USA; 4 Department of Surgery, Division of Plastic Surgery, Brigham and Women’s Hospital, Harvard Medical School, Boston, MA 02115, USA; 5 Department of Plastic, Aesthetic, Hand and Reconstructive Surgery, Hannover Medical School, 30625 Hannover, Germany; 6 Faculty of Informatics and Data Science, University of Regensburg, 93053 Regensburg, Germany; 7 Department of Plastic Surgery and Hand Surgery, Klinikum Rechts der Isar, Technical University of Munich, 81675 Munich, Germany; 8 Department of Plastic, Reconstructive, Hand and Burn Surgery, Bogenhausen Academic Teaching Hospital Munich, 81925 Munich, Germany

**Keywords:** facial palsy, facial paralysis, House-Brackmann scale, artificial intelligence, deep learning, bell’s palsy, smile restoration, facial reanimation, application

## Abstract

Background: The grading process in facial palsy (FP) patients is crucial for time- and cost-effective therapy decision-making. The House-Brackmann scale (HBS) represents the most commonly used classification system in FP diagnostics. This study investigated the benefits of linking machine learning (ML) techniques with the HBS. Methods: Image datasets of 51 patients seen at the Department of Plastic, Hand, and Reconstructive Surgery at the University Hospital Regensburg, Germany, between June 2020 and May 2021, were used to build the neural network. A total of nine facial poses per patient were used to automatically determine the HBS. Results: The algorithm had an accuracy of 98%. The algorithm processed the real patient image series (i.e., nine images per patient) in 112 ms. For optimized accuracy, we found 30 training runs to be the most effective training length. Conclusion: We have developed an easy-to-use, time- and cost-efficient algorithm that provides highly accurate automated grading of FP patient images. In combination with our application, the algorithm may facilitate the FP surgeon’s clinical workflow.

## 1. Introduction

Facial palsy (FP) presents with a varying symptom complex attributable to an array of etiologies [1,2,3,4,5]. FP annually affects up to 53 cases per 100,000 population yielding comparable incidence rates across biological sexes [6,7,8,9]. Most FP patients are diagnosed with idiopathic FP (Bell’s palsy) followed by trauma, viral infections, and tumors [10,11]. Predisposing factors in FP include, for example, hypertension, diabetes mellitus, inflammatory neural demyelination, and migraine [12,13,14,15]. The age classes between 45–55 years are particularly prone to develop FP [16]. The sequelae of FP encompass adverse effects on physical, psychological, and social levels. Due to interrupted or erroneous orchestration of mimic musculature, FP patients encounter flaccidity or synkinetic facial mass movements, respectively [17,18]. Micro- and macroanatomical studies have identified key muscles in FP pathology, such as the depressor anguli oris (DAO), the depressor labii inferioris (DLI), and the zygomaticus major muscles [19,20,21,22,23]. The malfunction of such muscular cornerstones leads to a disfiguring facial appearance and dysfunctional mimic movements [10,24]. Emotional expressiveness is hindered and smile symmetry is impaired [5,25]. The pathognomonic attributes of FP catalyze the manifestation of psychosocial disorders, including anxiety and depression [26]. Tseng et al. demonstrated that FP patients were 59% more likely to develop an anxiety disorder, as compared to unaffected individuals [27]. A 2016 South Korean study found that 32% of FP cases experienced ≥2 weeks of depressed mood versus 13% in the general population [28]. Further, increased levels of distress have been observed in FP patients [29]. In a vicious circle, such conditions promote social withdrawal and isolation as well as reduced quality of life [30].

Given the heterogeneous etiology and pathology of FP, only a few general recommendations in FP therapy with a sufficient body of evidence exist. For example, studies recommend the prescription of oral steroids to target acute FP cases [31,32,33]. The surgical management of FP symptoms ranges from free versus regional muscle transfer to (micro-)surgical techniques, including direct neurorrhaphy and neurotization procedures [34]. For specific indications, even further complex reconstructions have been proposed. Boahene et al. popularized the concept of multivectoral muscle flaps to account for specific human smile pattern, while Klebuc et al. described the DAO-DLI-transfer to address a hypertonic DAO in conjunction with a hypofunctional DLI [35,36]. Azizzadeh et al. have underscored the beneficial effects of modified selective neurectomies to address synkinetic facial musculature counteracting the natural smile [17]. If a patient’s eligibility for each surgical technique is critically reviewed and tailored on a case-by-case basis, FP surgery may pave the way for sustainable outcomes.

In each FP case, the grading of the disease severity is crucial to launch appropriate treatment strategies early on and evaluate the course of the FP in follow-up visits. Introduced to the FP community in 1985, the House-Brackmann scale (HBS) has been representing the standard classification system in FP diagnostics across different (non-)surgical specialties [37,38,39,40]. The overlaying of evidence-based clinical grading systems and state-of-the-art electronic facial recognition software carries promising potential for objective classification of FP disease [41,42]. However, there is a scarcity of step-by-step tutorials outlining the concrete steps that enable FP surgeons to successfully apply machine learning (ML) techniques in their patient work. We, therefore, aimed to develop an automated facial palsy grading system for FP surgeons interested in ML.

## 2. Materials and Methods

### 2.1. Data Acquisition from Facial Patients 

**Figure 1:**
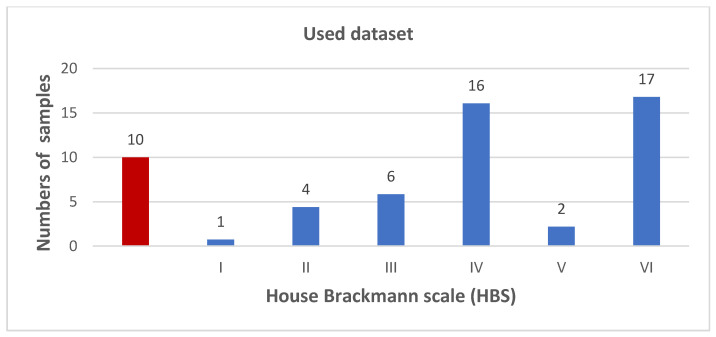
Overview of the study population and distribution of the House-Brackmann scale (HBS). The red bar is visualizing ten healthy individuals as a control group. Facial palsy (FP) patients with HBS scores of IV and VI accounted for the majority of cases, respectively.

From June 2020 to May 2021, prospective data acquisition was performed on 51 patients and additional 10 healthy patients as a control group seen at the Department of Plastic Surgery at the University Hospital Regensburg, Germany (Figure 1).

Inclusion criteria comprised a pathological HBS (i.e., >I) [40]. Of note, the HBS classifies FP severity levels from I (i.e., normal facial function) to VI (i.e., complete FP). Classification is conducted utilizing nine facial expressions (i.e., face in repose; raising the eyebrows; smile with mouth closed; full-denture smile; pursing the lips; gentle eye-closure; forced eye-closure; wrinkling the nose; depressing the lower lip). Facial expressions were recorded based on previous work by Volk and Hadlock [43,44]. As recommended by the Jena facial palsy group, patients were asked to perform these expressions to the best of their ability three times prior to photo documentation [43]. Photo documentation was conducted by either the first or last author (L.K., A.K.) during the last author’s facial palsy consultation hours utilizing the CANON EOS 400D with the respective flash unit (Canon, Ota, Japan). The examiner who did not take the patient photos supervised the documentation process. Prior to our first patient photo documentation, we consulted the clinical-intern photo department to evaluate our camera/photography settings. All patient photos were taken in the same examination room at the same spot to ensure a standardized camera distance. We further used a camera tripod with fixed setting sizes for standardized documentation. In cases in which patients were unable to perform the movement, the authors photographed the best attempt. In cases in which patients stated that they were not used to this facial movement and did not know how to perform the movement, the authors provided the same short instruction on how to theoretically perform the respective movement throughout all cases.

**Figure 2:**
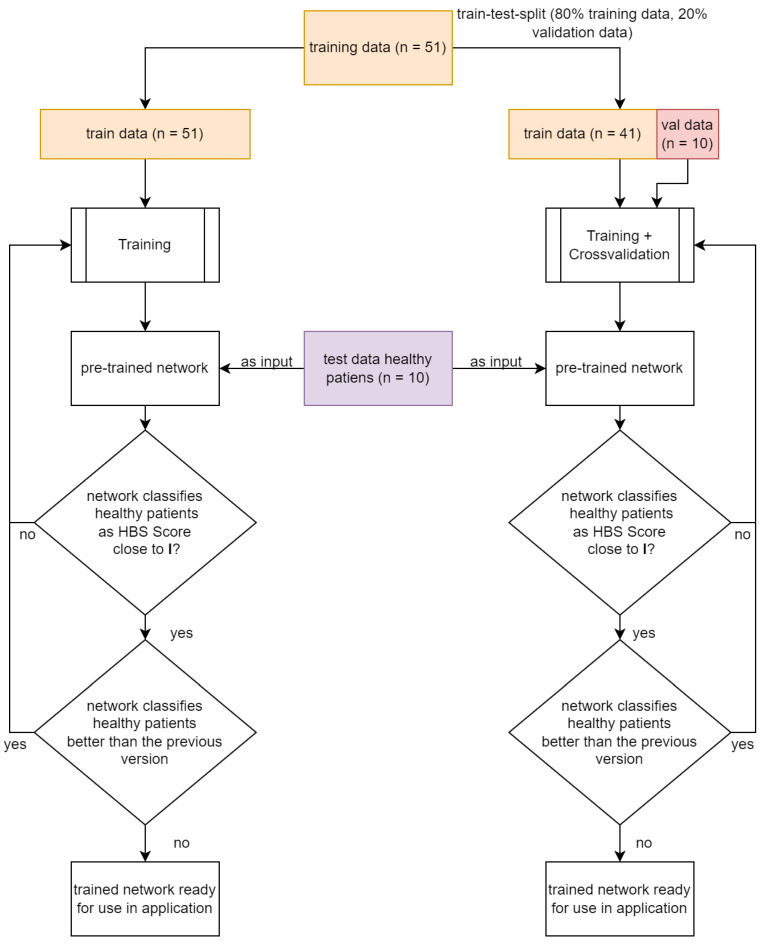
Flow chart of the workflow of how to train the network (training with and without cross-validation) with the training data and validate with healthy patients.

We included 51 patients and could therefore validate the network with ten patients since the dataset was divided into a training group with 41 patients and a validation group with ten patients. Of note, there is a difference between the ten patients with FP who were selected from the training data including 51 FP patients by means of a train-test-split and the ten healthy patients who were used for the final validation. The training/validation workflow is illustrated in Figure 2.

### 2.2. Facial Palsy Image Segmentation

We designed a facial palsy (FP) image segmentation method as the preprocessing section of the House-Brackmann score classifier, to automatically combine nine input images into one image. Each single image represents a certain facial expression. The nine images serve as input for the neural network, while the House-Brackmann scale (HBS) represented the output value of the network. Beforehand, the images had been pre-classified accordingly by three physicians specialized in FP therapy to set up a distinct link between the nine images and the corresponding HBS. The workflow is illustrated in Figure 2.

**Figure 3:**
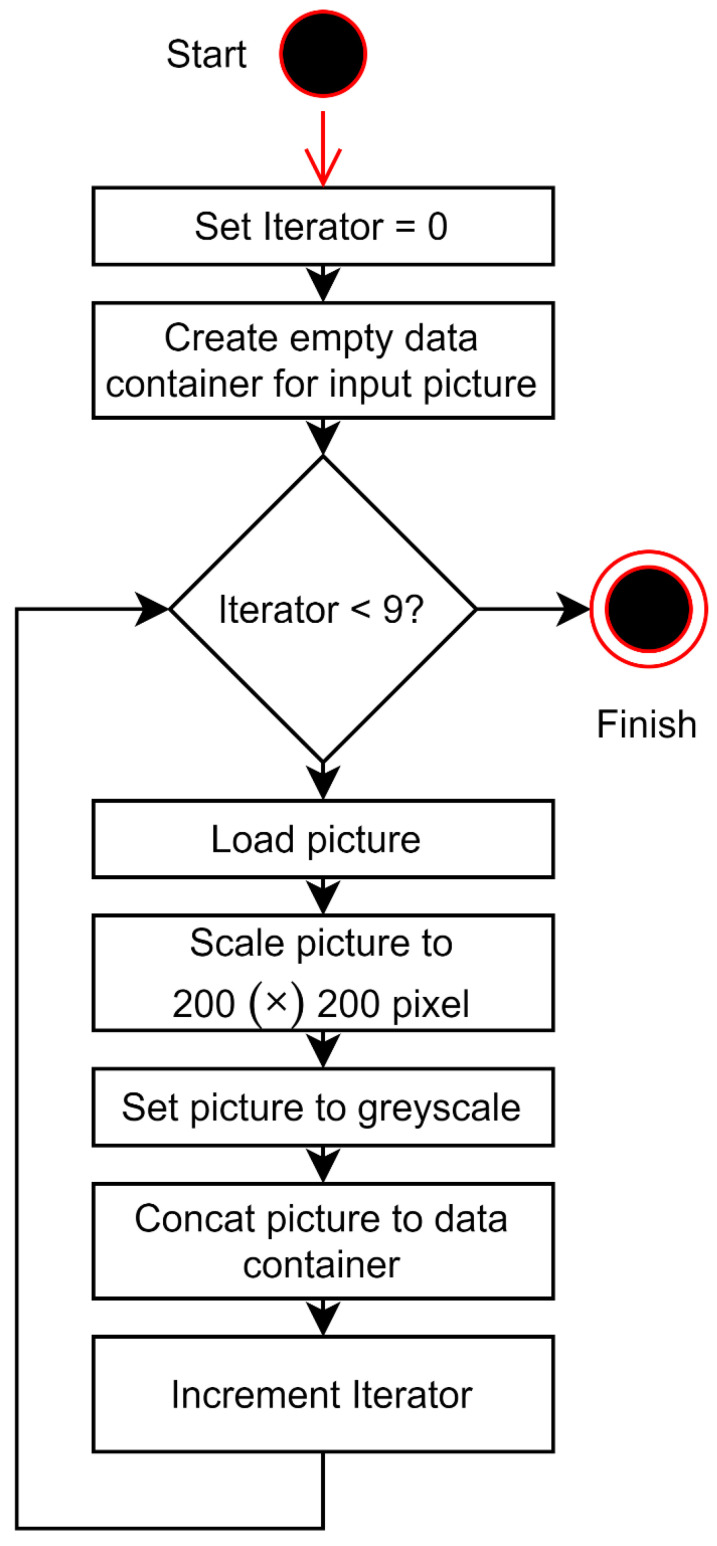
Preliminary image preparation. Transformation of nine single pictures to a single composed picture.

**Figure 4:**
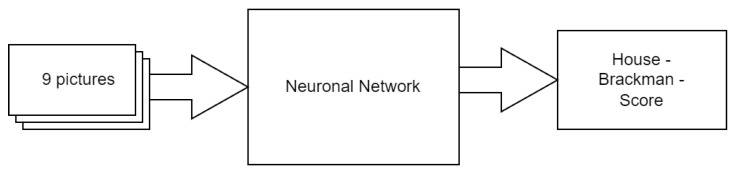
Basic workflow steps. Each patient image was assigned a distinct House-Brackmann scale (HBS) before being added to the neural network.

Due to the enhanced accuracy of the neural network, and with regard to its possible application in clinical situations, six individual outputs were chosen, each representing one distinct level in the HBS. First, the nine different patient images were implemented in a black–white format and scaled to 200 × 200 pixels to rationalize the computationally intensive training of the neural network. To adapt the nine colored patient images of arbitrary resolution to these requirements, an algorithm was utilized. The mesh yielded 200 × 1800 (Figure 3). The second step is the transformation of nine single pictures to single-composed picture input signals corresponding to the pixels of the nine patient input images and the six output signals, each representing one distinct level in the HBS. Concerning the output signals, each could either have a value of zero or one. For example, an HBS = VI should result in the output value = 1 for the VI. signal, whereas the output values = 0 for the I.–V. signals (Figure 4).

The neural network training comprises a set of patient images assigned with the corresponding HBS. Each row in the training set, therefore, corresponds to one patient. For training purposes, the data was stored in two arrays with one array for the input and one array for the output data [45].

### 2.3. Structure of the HBS Score Classifier 

**Figure 5:**
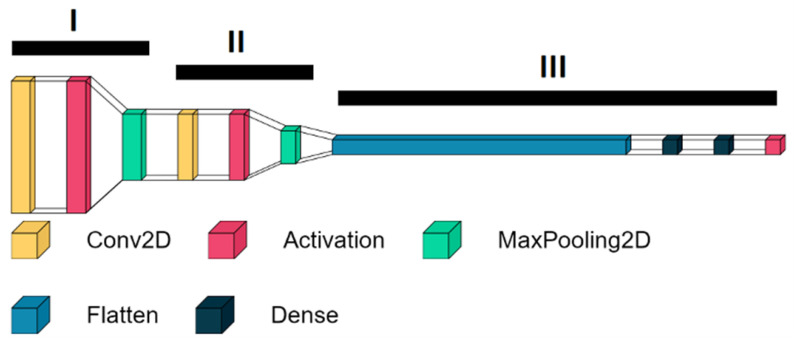
The different components of the machine learning model. The network is subdivided into three parts (i.e., I, II, III).

For the inner structure of the network, a multi-layer network with three parts was employed ((Figure 5) using machine learning models I, II, and III). The first two layers consist of a convolutional layer, an activation layer including the activation function “relu”, and a max-pooling layer. A convolutional layer is a layer in which several neurons are addressed. This enables a more general evaluation of inserted information. This layer can recognize and extract individual features from the input data [46]. A max-pooling layer is used to reduce the computational workload to allow for more efficient processing. Groups of inputs are mapped to individual neurons of the max pooling layer [47]. The activation function “relu” corresponds to the following equation:

$$f\left(x\right)=\{\begin{array}{c} 0\;if\;x<0\\ x\;if\;x\;\geq 0 \end{array}$$

This function is resource efficient and therefore matches the high throughput of data at the starting point of the neural network.

The classification process is conducted within the convolutional layer and the activation layer, while the max-pooling layer further refines the output, saves computing time, and prevents overfitting by excluding insufficient results. Overfitting leads to an overfitting neural network and occurs when the neural network is trained for too long with the training data, and therefore noise and random outliers in the training data are also adopted as a concept of the model. The problem is that such a trained network can no longer predict new data unknown to it. The size of the three stages is getting continuously smaller in the direction of the output. The output of the second stage is then filtered by a layer of flattening, which connects the second stage with the last stage. The last stage consists of layers with 64 and six neurons, respectively, with each neuron assigned to a distinct level of the HBS. At the end of the classification process, there is an activation layer including the activation function “sigmoid,” which corresponds to the following equation:

$$f\left(x\right)=\frac{1}{1+e^{-x}}$$

Since the results of this function can be between zero or one, this equation is commonly used as a transfer function in the output layer of neural network models to predict probabilities between 0–100%.

For training purposes, 80% of the patient data was used to train the network and the remaining 20% was utilized to validate the neural network. This is called cross-validation. The network underwent varying numbers of training epochs. During each epoch, stochastic gradient descent is used to best configure the neural network to map the input data (i.e., the patient images) and the output data (i.e., the predicted HBS). Following each training run, the network was retested to assess its prediction performance on previously unknown patient data.

Computer operations were performed in the Python programming language (version 3.10.2; Python Software Foundation, Beaverton, OR 97008, USA) on a Lenovo Thinkpad computer (T470, Intel Core i7–7600U processor running at 2.8 GHz with 32 GB of RAM and a Nvidia GeForce GTX 1650 Ti graphic card; Lenovo Deutschland GmbH, 70563 Stuttgart, Germany).

## 3. Results

### Number of Training Runs Determines Prediction Accuracy

Regarding the accuracy rate, 30 training runs proved to be the most effective. The average time of each training run was 9.6 h on our test machine. 

The performance of a neural network can be determined using the loss function. This is calculated as follows:

$$L\left(a_{i,}y_{i}\right)=-\left(y_{i}\log\left(a_{i}\right)+\left(1-y_{i}\right)\log\left(1-a_{i}\right)\right)$$

In this case, the loss function is used for binary classification, so the output can be zero or one. More precisely, one speaks of the “binary cross entropy loss” function. The index *i* always refers to the training examples. In the corresponding application, the network was trained with 51 patients and nine images were used to validate the network. The index *i* is therefore 51. Since it is a binary function, the result can only be zero or one. This calculation then leads to the loss or validation loss of the trained neural network.

After training the network, we had a loss of 0.49 for the training data and a loss of about 0.83 for the validation data. The accuracy for the training data and the validation data was 80% and 52%, respectively.

When training without validation, i.e., using all available patient images without using cross-validation, an accuracy of about 98% was achieved with a loss of less than 0.1. This showed that a longer training of >100 epochs was necessary. After training, the algorithm processed the real patient image series (i.e., nine images per patient) in 112 ms.

Overall performance could be improved by using more training data. Another point of leverage includes adapting the network architecture. To this end, more layers could be added. Further, the resolution of the input data (currently 200 × 1800 pixels) could be increased. This would render the prediction more independent of physical characteristics, such as beard growth or skin color, which can currently still impair algorithm predictions. Ideally, patients should be asked to remove any coverings, such as hair and/or any other body modification prior to photographic documentation. Another optimization method involves deepening the network structure. Currently, the network consists of three calculation levels, while more calculation levels could be integrated here. The use of non-sequential neural networks (i.e., the insertion of parallel computation strands into the network) can also enhance network performance. This approach is based on the concept that the network can then simultaneously compute different tasks with different resolutions, meaning that it can detect different templates in the input data.

**Figure 6:**
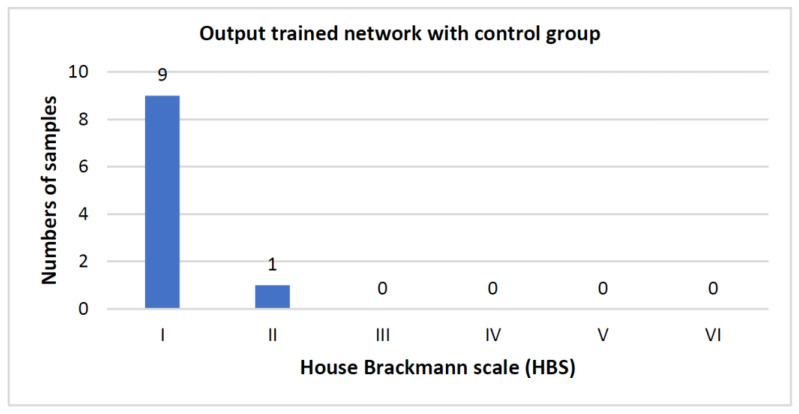
Evaluation of the control group. The control group comprised of ten healthy individuals of whom only one was assigned a pathological House-Brackmann scale (HBS) score (i.e., HBS > I).

To test the trained network, data from a healthy control group was used. As the network was only trained with FP patients, the results were expected to be close to an HBS of one. Ten healthy individuals were used as a test group. The results of the control group are shown in Figure 6. Only one individual was assigned a pathological HBS score (i.e., HBS > I) resulting in a false positive rate of 10%.

To visualize the results of the neural network, an application was coded that implemented different states (i.e., “Init”, “Waiting”, “Ready”, “Error”, and “Run”). The workflow of the application is summarized in Video S1.

**Figure 7:**
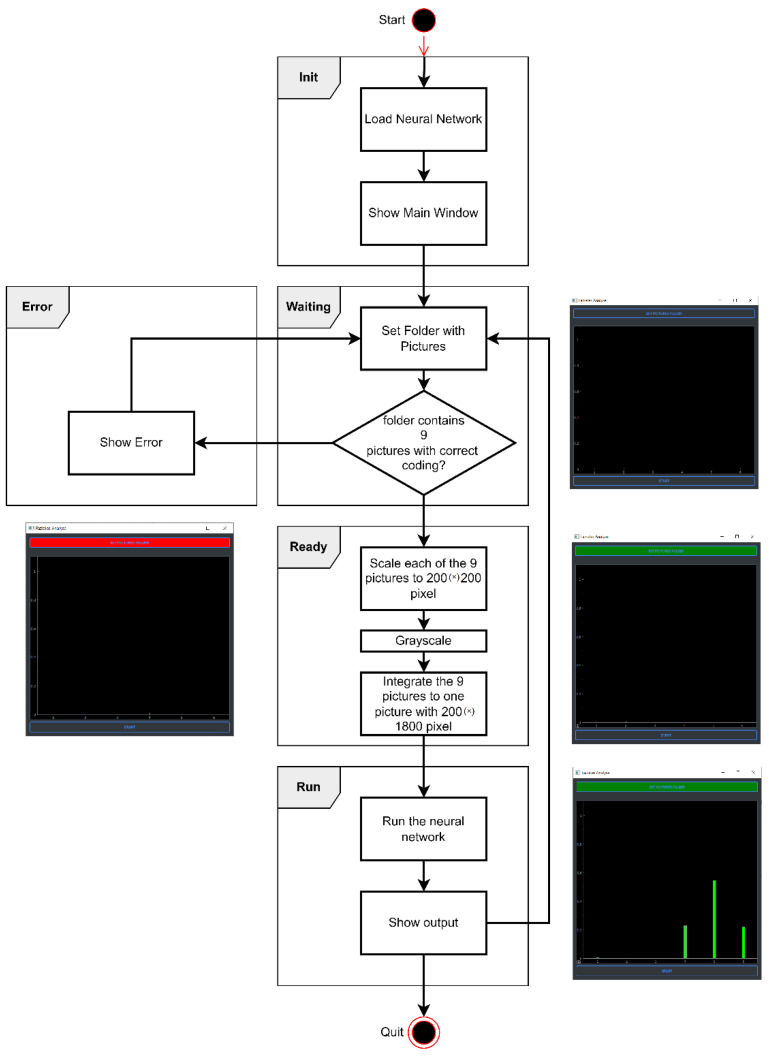
Exemplary application run. Sample output of the network utilizing the application.

First, the trained neural network is loaded in the “Init” state. When the nine patient images with the correct coding for the corresponding nine facial expressions are not completely available in the selected folder, the program switches to the “Error” state. The user can return to the “Waiting” state by selecting a correctly filled folder and then proceed to the “Ready” state in which the images are processed according to the aforementioned settings (i.e., black–white format; 200 × 200 pixel resolution). In the “Run” state, the processed images encounter the mesh. The output of the network is displayed as a bar chart. Here, each bar corresponds to the output value of each output neuron of the network (Video S1). Figure 7 illustrates the process workflow of the application.

## 4. Discussion

The ever-increasing challenging work environment has resulted in one-third of reconstructive surgeons and surgery residencies reporting burnout symptoms [48]. Yet, recent studies have predicted a future shortage of 3000 US reconstructive surgeons by 2050 and calculated that about 25 million US people have insufficient access to reconstructive surgery services, meaning that a decimated surgery workforce will soon face an increasing work volume [49,50]. This exemplary discrepancy underscores the relevance of time- and cost-efficient tools that facilitate the FP surgeon’s workflow. ML has demonstrated beneficial effects in clinical applications, such as in the postoperative monitoring of free flap viability based on skin color or the identification of melanomas using smartphone images [51]. In this study, we provide a time-efficient, user-friendly, and cost-free FP grading algorithm.

In the senior author’s experience, thorough grading of FP patients based on the most commonly used classification system, the HBS, can take up to five minutes or even longer in complex FP patient subsets (e.g., neurofibromatosis or apoplex patients). It is not unusual for FP specialists to examine 30–40 FP patients per day, which might accumulate to several hours of grading per day. While these numbers represent worst-case scenarios, the time-saving potential of automated FP grading is indisputable. Further, additional diagnostic tools, such as ultrasound imaging, have gained popularity in FP examination [52,53,54]. To include such diagnostic add-ons into the packed clinical routine, FP surgeons first must save time on other tasks such as FP grading. Utilizing our algorithm, we could process real patient image series (i.e., nine images per patient) in 112 ms, on average, which is comparable to the elegant approach developed by Haase et al. (108 ms) [55]. Our model requires only nine standardized patient images, whereas comparable systems have to be fed with video content longer than 20 min per patient [56]. Given the structured simplicity of our model, the entire grading process could be assigned to technical assistants, saving the FP surgeon additional work time and allowing for more time spent on direct patient-doctor communication which has been shown to decrease decisional conflicts and preoperative anxiety from the patient’s side [57]. Morrell et al. demonstrated that even five minutes of extra doctor-patient time significantly improved patient satisfaction with their medical provider [58]. From the surgeon’s side, such patient-doctor interaction can counteract burnout symptoms and promote work satisfaction [59]. More precisely, repetitive and routine tasks, such as systematic grading, have been identified as burnout drivers, including the recommendation of experts to outsource such work to robotic/computerized assistance tools [60]. Our algorithm may allow for a more refined and self-defined time allocation among the FP surgery workforce.

Recent efforts have focused on combining ML and 3D-frameworks to detect, for example, volume deficits caused by long-term facial musculature atrophy in FP patients [61]. By implementing such techniques, providers aim for advanced grading, ultimately leading to a more differentiated decision-making process in FP therapy [62]. The link between ML and 3D-techniques has resulted in the development of different networks such as AlexNet. Since its launch in 2012, AlexNet has been successfully used in a broad medical application field (e.g., to detect pathologic MRI brain scans or to classify chest X-rays of COVID-19 patients) [63,64,65]. Based on the HBS, Storey et al. have programmed the 3DPalsyNet, which yielded a classification accuracy of up to 86% (vs. up to 99% in our model). Their algorithm had poor accuracy levels when grading more difficult FP images [66]. Other comparable networks have shown accuracy scores ranging from 88 to 97% [64,66,67,68]. Zhao et al. demonstrated the prognostic value of a 3D dynamic quantitative analysis system in acute FP cases. However, for each case, the examiner must position six cameras in front of the patient so that every reflective point on the patient’s face is detected by at least three cameras [69]. Such preliminary work increases the overall examination time per patient, whereas our platform demonstrated accuracy levels of 99% on images taken with a standard camera widely available in the hospital setting. Anecdotally, the set-up and positioning did not take longer than one minute for our model. Of note, our network can also process images taken with modern smartphones, which may further promote cost-effectiveness. The concept of 3D-technology linked to ML is intriguing, although consequent advantages of such joint systems in grading accuracy when compared to 2D-based platforms remain to be ascertained. Due to their complex and multi-layer neural architecture, such platforms require an extensive and cost-intensive hardware fundament as well as maintenance and acquisition costs of up to $49,000 [70,71]. Advanced programming skills far beyond the FP surgeon’s scope are oftentimes needed to develop (and use) such joint systems [72]. Another study by Jiang et al. also involved a highly precise automated grading concept in FP patients. Their work focused on measuring facial skin microcirculation perfusion distribution in FP patients [67]. The Jena group proposed an FP grading index prediction model by using the eFace grading index, which features 16 ordinal fine-grained grading scales for resting face and facial motions [68,73]. The authors addressed objective FP assessment as a linear regression problem instead of an index classification method given the finely graduated ordinal sub-scales of the eFace-scale. Their dataset included image series of 52 multi-ethnical patients of different ages before and after undergoing a hypoglossal-facial anastomosis. Each image series contained nine standardized images of the patient’s frontal face. In a second dataset, they included 28 adult healthy subjects as a control study. The authors reported a mean absolute error (MAE) of 11% in FP patients versus 12% in the control group. The MAE might be further reduced by enlarging the study sample. They also found that deeper networks, such as ResNet-50, did not provide more suitable features for their application, while containing more parameters than a standard VGG-16 model in case fully connected layers were excluded. The authors further outlined the potential adaptation of this approach to be used in other FP scales, such as the Sunnybrook facial grading system [74]. Another study from the Jena group introduced an automated FP grading system based on the Sunnybrook facial grading system [75]. To this end, the authors used 4572 photographs of 233 patients with unilateral peripheral FP. They reported an intraclass coefficient of 0.35 comparing subjective and objective/automated FP grading. The implementation of the Sunnybrook facial grading system carries high translational potential for clinical use, given the recommendation of the Sir Charles Bell Society to use the Sunnybrook facial grading system as a standard grading system for reporting outcomes of facial nerve disorders [76]. Gaber et al. used the Microsoft’s Kinect (v2) for real-time FP grading [77]. Their approach was based on the detection of facial landmarks as 3D coordinates both for resting symmetry and voluntary movements, such as raising eyebrows or smiling. Calculation of the regional facial asymmetry was performed through the ratios of distances between corresponding landmarks and a common reference point on the two sides of the face. They also included gamma correction, as well as eye area and mouth slope features. Their system was tested on healthy individuals and showed promising results, yielding a symmetry index of 98% for the ocular region and 96% to 99% for the oral region. A 2017 study by Guo et al. suggested the use of deep convolutional networks for objective FP grading based on the HBS [78]. The authors addressed the problem of confusing neighboring HBS degrees by refining the GoogLeNet model resulting in a classification accuracy of 91% for predicting the HBS degrees. Their dataset included 105 FP subjects versus 75 healthy subjects. Each image set contained four different facial expressions totaling 720 labeled images. Interestingly, the authors designed a data augmentation step to account for the imbalance in HBS degree distribution. Data augmentation included horizontal flipping, random rotating, and resizing, as well as adding salt and pepper noise.

**Figure 8:**
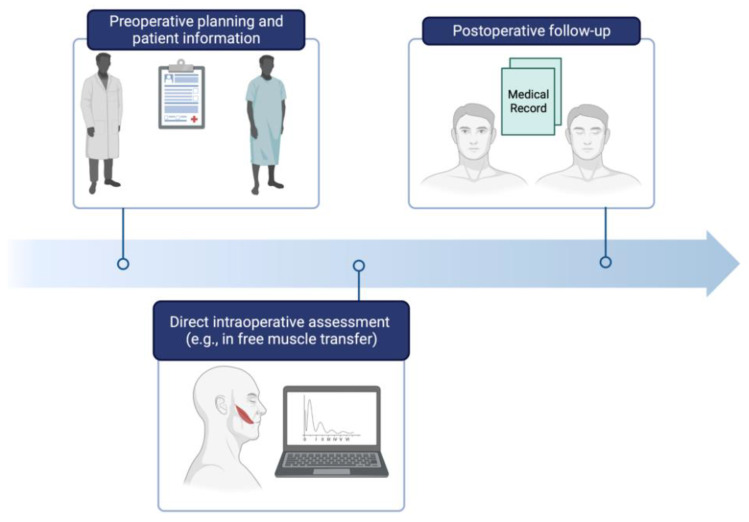
Implementation of automated grading in the clinical workflow. Automated grading could be used in the preoperative planning phase, as well as for direct intraoperative assessment. Following (non-)surgical therapy, automated grading may allow for standardizing patient follow-up evaluation.

We propose a simple, yet easy-to-use application that allows FP surgeons with varying informatic knowledge to directly utilize our model. With the recent advancements in 3D-technology being promising, we are looking forward to including this innovative technique into our model, as soon as the barriers of cost-effectiveness, user-friendliness, and time-consuming preliminary work have been overcome. Together with other imaging techniques, such as ultrasound or MRI, this approach might enlarge the FP surgeon’s diagnostic arsenal and allow for comprehensive patient evaluation at different time points of FP therapy (Figure 8) [54].

### Limitations

The present study is not without limitations. Our study population comprised a disproportionate percentage of severe FP cases. To account for this imbalance, we performed oversampling. We included 51 patients in this study. Therefore, large-scale studies are needed to corroborate our findings and demonstrate the efficiency of our algorithm in larger patient cohorts. However, our study population did accurately represent the most common clinical FP scenarios. The HBS represents the most commonly used FP grading classification system in US clinics but has revealed certain downsides such as the insufficient implementation of synkinesis [79]. Thus, we aim to translate the algorithm into more sophisticated grading systems, such as those developed by Guarin and Hadlock [41,80,81]. Work done by the Jena group underscored the implementability of automated grading approaches into the Sunnybrook facial grading system [75]. The study by Guo et al. provided further potential points of leverage to target the imbalance of HBS degree distribution [78], while our study demonstrated the general feasibility of combining all photos to generate one single score. Yet, further efforts are needed toward creating a tensor with the nine images per FP patient instead of combining the images which can cause dilution of the information present in the images. 

## 5. Conclusions

We have developed an easy-to-use, time- and cost-efficient, as well as highly accurate algorithm utilizing ML principles. Integrated into a user-friendly application, our model may facilitate and accelerate the FP surgeon’s clinical workflow. 

## Data Availability

Not applicable.

## Supplementary Materials

The following supporting information can be downloaded at: https://www.mdpi.com/article/10.3390/jpm12101739/s1, Video S1: Exemplary application run. The simple, easy-to-use, working surface allows for an uncomplicated and time-efficient application running.
